# Vitamin D and Demyelinating Diseases: Neuromyelitis Optica (NMO) and Multiple Sclerosis (MS)

**DOI:** 10.1155/2020/8718736

**Published:** 2020-01-19

**Authors:** Cady Rodney, Sherriann Rodney, Richard M. Millis

**Affiliations:** Departments of Clinical Medicine and Pathophysiology, American University of Antigua College of Medicine, Antigua and Barbuda

## Abstract

Vitamin D deficiency is prevalent in all ages regardless of climate or geographical location and evidence is emerging that the incidence of autoimmune diseases is increasing worldwide. Women make up a large proportion of autoimmune disease diagnoses, underscoring the importance of fully elucidating the complex synergistic relationships between estrogens and vitamin D. Vitamin D receptor-activating drugs appear to enhance remyelination in patients diagnosed with multiple sclerosis (MS) and other demyelinating diseases such as neuromyelitis optica (NMO). This review is intended to update health practitioners about the potential role of vitamin D deficiency demyelination and to motivate future research on dietary recommendations for vitamin D in preventing and treating demyel1nating diseases.

## 1. Introduction

The incidence of autoimmune diseases continues to rise globally [1] while population vitamin D levels fall [2]. The role of vitamin D in the debilitating autoimmune diseases known as neuromyelitis optica (NMO) and multiple sclerosis (MS) is a subject of interest. NMO resembles MS in terms of clinical presentation and many cases of NMO are thought to be initially misdiagnosed as MS [3]. This review is intended to inform health practitioners about the immunological functions of vitamin D so that its role may be considered in the process of treatment. The additional aim is to also highlight similarities and differences between NMO and MS, with the intention of helping to reduce the incidence of misdiagnosis in the future.

NMO and MS are both immune-mediated demyelinating diseases targeting the CNS. Since the CNS has a limited and questionable regenerative capacity, restoring lost function is not always guaranteed for patients and most treatments are aimed at preventing future episodes (prophylaxis) and symptom relief as opposed to curative measures. Vitamin D, a hormone that humans endogenously produce and also consume, has long been the focus of many MS studies, but may require an innovative approach to yield beneficial therapeutic results.

### 1.1. Vitamin D—The Cause or Result of Demyelinating Disease?

Vitamin D is mostly known for its roles in calcium and bone homeostasis; however, it also contributes to nervous system development, blood-brain barrier maintenance, axon growth/regeneration, and myelination which are all important aspects to be considered in autoimmune demyelinating disease [4]. Further research is necessary to determine the true potential of vitamin D as the ultimate prophylaxis against these autoimmune attacks on the myelin sheath.

Research demonstrating correlations between vitamin D status, disability, and relapse rate for NMO has provided the rationale for vitamin D supplementation as a therapeutic and preventive strategy for this disease [5]. On the other hand, conflicting hypotheses suggest that vitamin D supplementation is not helpful and contraindicated because it may exacerbate the NMO disease process [6]. It, therefore, remains unclear whether vitamin D deficiency sets the stage for autoimmune attack or is the result of the pathophysiology underlying disease. It is known for sure, however, that there is a high prevalence of vitamin D deficiency among patients with NMO and MS due to external factors such as immobility, sun avoidance, and corticosteroids [7]. In the meantime, researchers and health practitioners alike should learn more and think about how vitamin D factors into both the genetic and environmental causes proposed for autoimmune demyelinating diseases. This includes vitamin D receptors, single nucleotide polymorphisms, and estrogen interactions which are discussed in this review.

### 1.2. Biosynthesis of Vitamin D

Exposure to UVB radiation converts an endogenous precursor, 7-dehydrocholesterol in skin to the animal form of vitamin D, cholecalciferol, also known as vitamin D3 (vitamin D2 is the plant form, ergocalciferol). Cutaneous penetration of ultraviolet radiation is a key step in the biochemical activation of human vitamin D. Thus, darker skin pigmentation increases the risk of vitamin D deficiency. Evidence is emerging that dark skin pigmentation is a risk factor for autoimmune disease; therefore, providing a logical linkage between these conditions and African ancestry [8]. Specific vitamin D receptor (VDR) polymorphisms (discussed later) have been identified in populations with darker pigments, and are associated with elevated risk of developing MS [9].

Before vitamin D can perform its functions, it is activated first by liver 25-hydroxylases (to 25(OH)D) and then by kidney 1*α*-hydroxylases to become 1,25-dihydroxy vitamin D (1,25 (OH)D), also known as calcitriol. According to the Food and Nutrition Board [10], the best clinical marker to determine vitamin D levels is 25(OH)D since it has a longer half-life (approximately 15 days) compared to 1,25(OH)D which has a shorter half-life (approximately 15 h) [11]. 25(OH)D is a measure of both the vitamin D consumed from food/supplementation and endogenously (by the skin), but does not indicate amount in tissue storage, therefore only serving as a marker of exposure [10].

The actions of vitamin D3 are produced by a signaling cascade initiated by calcitriol binding to the nuclear vitamin D receptor (VDR) which acts as a transcription factor. Calcitriol forms a heterodimer with the retinoid-X-receptor which also binds 9-*cis* retinoic acid, a form of vitamin A known for its functions in the Wnt stem cell signaling pathway (an important regulator of tissue development and carcinogenesis) [12]. The calcitriol-VDR heterodimer binds to hormone response elements on DNA resulting in expression or repression of specific gene products including those associated with inflammation [13]. The VDR is also involved in micro-RNA regulation of posttranscriptional events and epigenetic regulation of protein expression [14]. VDRs are found in the majority of human cells [15] calling for more attention to be placed on the lesser-known effects of vitamin D, those other than in bone tissue. Evidence is emerging that VDRs are involved in the regulation of cardiovascular, metabolic, and immunologic functions [15]. As a result, vitamin D deficiency has been linked to other diseases besides those of bone origin including, but not limited to, cancer, cardiovascular diseases, and autoimmune diseases [16]. The following section discusses a putative protective role of vitamin D in the demyelinating diseases NMO and MS.

### 1.3. Vitamin D Protection against Autoimmune Diseases: Immunomodulation

**Figure 1:**
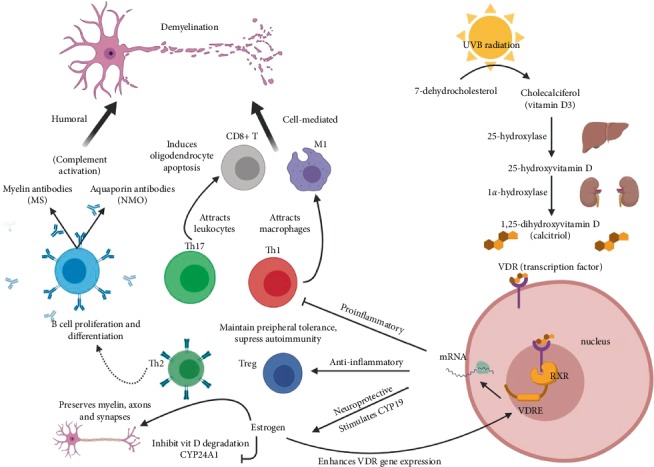
A roadmap of vitamin D production and putative roles in autoimmune demyelinating disease. Once activated to calcitriol by liver 25-hydroxylases and kidney 1*α*-hydroxylases, vitamin D can execute a variety of immunologic functions. Calcitriol binds the VDR receptor which acts as a transcription factor when complexed with nuclear retinoid X receptor. The VDR-RXR heterodimer then binds to DNA sequences within gene promoter regions (vitamin D response elements) and facilitates the expression of gene products which may be classified as neuroprotective, anti-inflammatory, and proinflammatory. Vitamin D increases estrogen production (stimulation of aromatase (CYP19) converts testosterone to estrogen). Estrogen preserves vital components of the CNS attacked in autoimmune disease and regulates vitamin D production (inhibits degradation by 24-hydroxylase (CYP24A1). Vitamin D suppresses autoimmunity by promotion of anti-inflammatory T cell subsets: T helper 2 (Th2) and regulatory T (Treg) cells. Vitamin D downregulation of proinflammatory cytokines IL-1 and IL-21 (not depicted) and T cell subsets: T helper 1 (Th1) and 17 (Th17) prevents macrophages and other leukocytes from exerting cell mediated toxic effects on myelin. B cell proliferation and antibody production are also inhibited, knocking out the key mediators of humoral mediated demyelinating damage. Th2 cells are pathologically involved in the induction of B cells producing self-antibodies in MS, but a higher ratio of Th2 to Th1 is associated with reduced inflammation. M1 macrophages release proinflammatory cytokines, metalloproteases and free radicals and carry out phagocytosis of myelin (early stage of MS) that leads to CNS destruction while M2 macrophages repair inflammatory damage (late stage of MS). In NMO, macrophages may scavenge the byproducts of astrocyte cytotoxicity and eosinophil/neutrophil infiltration (not depicted). *Created with *https://Biorender.com.

Vitamin D is more popularly known for maintaining appropriate calcium levels to support bone mineralization and less recognized for its immunomodulatory function. Cells of both the innate and adaptive branches of the immune system display 1*α*-hydroxylase activity and express VDRs [16]. Vitamin D suppresses both B cell proliferation and differentiation, thus regulating antibody production by plasma cells [16]. Vitamin D also affects T cell proliferation and maturation by inducing differentiation of naïve CD4+ T cells into immunomodulatory T helper 2 (Th2) and regulatory T (Treg) cells [5]. In turn, vitamin D decreases the production of proinflammatory Th1 and Th17 cells and downregulates production of interleukins (IL-1 and IL-21) and other proinflammatory cytokines [5]. Studies on experimental autoimmune encephalomyelitis (EAE), currently the best animal model of MS, have suggested that vitamin D exerts both prophylactic and therapeutic effects via these immunomodulatory mechanisms [16]. For a schematic diagram of these immunomodulatory functions and their possible role in pathology of NMO and MS, see Figure 1.

### 1.4. Immunopathology of MS

Multiple sclerosis is the most common autoimmune demyelinating disease [17], yet the initiating factor that leads to the disorder is still a mystery. It has been well investigated that the body's T helper cells play a major role in the unsheathing of neurons located in the CNS. Th1 cells activate macrophages by secreting the chemokine IFN-*γ*, whereas Th17 cells disrupt the blood brain barrier (an early event in MS) and attract other leukocytes to help in the attack against oligodendrocytes [18]. Oligodendrocytes produce myelin which insulates neurons of the brain and spinal cord specifically. Altogether these leukocytes cause damage to the protective fatty layer that allows for fast conduction of nerve impulses. Depending on the CNS site where lymphocyte toxic byproducts cause neuronal injury, there is corresponding neurological deficit. MS is characterized clinically by repeating subacute attacks on the CNS with severe impairment in central motor coordination (balance), sensory and cognitive deficits, optic neuritis, and fatigue [16]. Remyelination may or may not occur resulting in either spontaneous recovery or a lasting deficit respectively. Vitamin D may be the common link between genetic and environmental factors in the development of MS. Each copy of the MHC DRB1∗1501 allele inherited by an individual triples their risk of developing MS [19] and evidence is emerging that vitamin D modulates the effect of the HLA-DR15 allele on the onset of MS [13].

### 1.5. Immunopathology of NMO

Vitamin D may be a key mediator in NMO, an autoimmune disease which demyelinates the optic nerve, brain stem and spinal cord. Antibodies specifically directed against aquaporins (water channels) within astrocytes appear to be a key pathophysiological feature of NMO; however, the initiating factor remains unknown [20]. Identification of the specific antibody anti-aquaporin-4 (AQP4 antibody) has facilitated diagnosis of a spectrum of NMO-related disorders and has made a clear distinction between NMO and MS as separate diseases [20]. The true number of NMO cases may be higher than the 1/100,000 (Europe) and 4.4/100,000 (North America) reported due to a misdiagnosis rate of approximately 30–40% before AQP4 antibody testing became available [3]. Antibody activates complement production by the astrocytes of the blood brain barrier, increasing permeability and leading to inflammatory infiltrate. Astrocytes, oligodendrocytes and neurons are not the only targets of complement mediated destruction. Damage extends to vasculature within the CNS as complement membrane attack complex causes hyalinization of blood vessels [20]. T cells are indirectly involved in lesion development by inducing cytokine release necessary for antibody production and granulocyte recruitment [21].

The majority of NMO spectrum cases are characterized by relapsing attacks of optic neuritis and/or myelitis happening sporadically [3]. Fewer patients experience a monophasic course in which attacks happen for a limited time span of days or weeks with no subsequent occurrences [3].

### 1.6. Similarities & Differences between NMO and MS

From an immunological standpoint, the cerebrospinal fluid of patients with MS contains all mononuclear white blood cells while NMO relapsing patients have polymononuclear components [22]. Unlike MS, the inflammatory infiltrate of NMO consists of eosinophils and neutrophils [20]. Oligoclonal IgG bands are also relatively rare in NMO compared to MS [21] since clonal expansion of B cells is less common [20]. Serum levels of memory Th17 cells, IL-17A (induces neutrophil attracting chemokine secretion by other cells) and IL-23 (prolongs Th17 survival and enhances function) are significantly increased in both patients with NMO and MS [23]. Th17 levels correlate with disability (as measured by the Expanded Disability Status Scale (EDSS)) and relapse frequency in both diseases [20]. Notably, these levels are higher in NMO patients and suggests more severe demyelination/inflammation [2], but as mentioned earlier, vitamin D downregulates Th17.

NMO spectrum disorders present with fatigue, pain, depression, and sleep problems, painting a very similar clinical picture to MS [24]. Relapses and recovery however are much more severe in NMO relative to MS [22]. In fact, there is a faster build up to irreversible neurological disability and poorer prognosis in NMO than MS [22]. Additionally, NMO has a greater prevalence in women [21]. Autoimmune disease such as systemic lupus erythematosus and myasthenia gravis may coexist with NMO but is a rare find with MS [21].

Treatment for NMO spectrum disorders and MS are both aimed at immunosuppression and still very experimental. Both utilize corticosteroids for acute exacerbations [24, 25], but the MS disease modifying drugs (DMDs) such as interferons can worsen NMO [22]. Vitamin D serum levels of patients with MS have been of interest and supplementation has had mixed results as is discussed further below. NMO patient vitamin D levels have been investigated [5] to a lesser extent; however, the effects of supplementation have been reported in few studies.

### 1.7. Vitamin D Serum Levels in Patients with NMO and MS

Vitamin D plays a vital role in immunity, reflected in the low serum vitamin D levels in patients diagnosed with various autoimmune diseases [26] including MS [27, 28] and NMO [5, 29, 30]. Clinical significance of Vitamin D deficiency is demonstrated by a significant correlation between vitamin D levels and disease disability [5].

Significantly lower vitamin D levels in NMO patients relative to healthy controls has been reported with an inverse correlation between vitamin D levels and disability status score [5]. However, this study failed to find any difference between blood levels of vitamin D at relapse or remission, nor any association between vitamin D levels and annualized relapse rate. Another study found that while vitamin D deficiency was common in NMO patients from a Thai population, no correlation was apparent with disease activity or disability [30]. Ascherio et al. [28] were able to detect a 57% lower formation of new active lesions and 57% lower relapse rate when serum vitamin D levels of MS patients were higher, suggesting a possible predictive role in terms of progression rate. Kragt et al. [27] discovered that for every 10 nmol/L increase in serum vitamin D level, the odds of MS in female patients appeared reduced by 19%. These results suggest a “protective” effect of higher vitamin D serum levels and presented a negative correlation between Expanded Disability Status Scale and vitamin D levels [27].

Myasthenia gravis (MG) is another autoimmune disease that may clinically present similar to MS and NMO (and may coexist with the latter), but antibodies target postsynaptic acetylcholine receptors of the neuromuscular junction. Askmark et al. [31] reported significantly lower plasma vitamin D levels in patients compared to healthy controls and found supplementation beneficial as it improved fatigue score in patients with MG by as much as 38%. It was therefore proposed that monitoring of vitamin D3 was a vital parameter in patients with MG and supplementation required when levels are low as both a prevention and an adjunctive treatment strategy [31]. The following section discusses the outcome of vitamin D supplementation in NMO and MS.

### 1.8. Vitamin D Supplementation in Clinical Trials for NMO and MS

It is currently recommended that adults consume at least 600 IU of vitamin D daily to achieve adequate serum levels of 25-hydroxyvitamin D (25(OH)D) greater than 75 nmol/L [32]. Fish oil, animal protein, fortified dairy and cereal products may be sufficient dietary sources for adults free of disease [32]. Deficiency is suggested by serum levels less than or equal to 50 nmol/L [2].

According to a study by Burton et al. [33], a normal vitamin D level may not be sufficient to ward off MS and higher levels are required to observe immunomodulatory effects. This clinical trial utilized high doses of vitamin D (as opposed to low doses of vitamin D or calcitriol in earlier MS studies) and saw significant decreases in T cell reactivity and proliferation. Notably, despite giving doses of approximately 10,000 IU/day to patients over a 52 week period (corresponding to serum levels of >400 nmol/d) there were no reported adverse biochemical or clinical events, including hypercalcemia. But even with serum levels way above physiological range, the cytokine profile of pro-inflammatory and anti-inflammatory markers did not reveal any pattern. Researchers pointed out that changes may have occurred too quickly or too small of an extent to be detected [33]. Another study by Mahon et al. [34] failed to show any noteworthy increase or decrease in cytokines (such as TNF-*α*, IFN-*γ*, and IL-13) with vitamin D supplementation although slight reductions in IL-2 mRNA levels. Mosayebi et al. [35] administered doses of 300,000 IU/month of vitamin D3 to MS patients over 6 months and were able to find decreased cell proliferation and increased anti-inflammatory markers: TGF-*β* and IL-10, relative to the control group. The study was not able to identify a significant difference however between treatment and control in terms of expanded disability status score and gadolinium enhancing lesions. Soilu-Hänninen et al. [36] raised the 25(OH)D serum levels of patients with MS from a mean of 54 nmol/l to 110 nmol/l. Results indicated fewer new T2 lesions, a significantly lower number of T1 enhancing lesions, reduced disability accumulation and improved timed tandem walk but no effect on relapse rate [36]. Kampman et al. [37] observed no significant differences in annualized relapse rate (ARR), expanded disability status scale scores, multiple sclerosis functional composite (MSFC) components, grip strength, and fatigue despite MS patients having serum levels of 121 nmol/L after supplementation (20,000 vitamin D3 IU/week). A case report from Iran demonstrates a significant decrease in IgG-NMO titers in two patients after 15 weeks of high doses of vitamin D supplementation of 50000 IU of per week [29]. The mixed outcome of supplementation even with high doses makes the contribution of vitamin D questionable in MS and possibly NMO.

To uncover the potential benefits vitamin D may have in treatment of NMO, MS and other autoimmune diseases, its mechanism of action as an immunomodulatory agent must be investigated more thoroughly. This review identifies epigenetic modifications, VDR polymorphisms, and estrogen interaction as potential avenues for further study.

## 2. Epigenetic Modifications and VDR Polymorphisms

Glycosylation is shown to be an important epigenetic modification of the immune response [38]. A study by Grigorian et al. [39] in mice demonstrates how N-glycosylation may be involved, by a vitamin D-related mechanism, in the pathogenesis of MS. Mice employ an N-glycan branching pathway that appears to prevent the neurodegeneration seen in this autoimmune disease and if deficient in the N-glycan pathway, they display T cell hyperactivity and late onset of spontaneous inflammatory demyelination [39]. This glycosylation pathway also regulates the surface expression of CTLA-4 (an immune checkpoint receptor) by inhibiting endocytosis and therefore promotes growth arrest [40]. Aside from MHC loci (discussed earlier), genome wide association studies have identified a role of single nucleotide polymorphisms IL-2 (*IL2RA*) and IL-7 (*IL7RA*) *α*-chain receptor genes in MS [41]. The variants *IL7RA∗C* and *IL2RA∗T *downregulate the protective N-glycan branching pathway by inhibiting expression of *MGAT1, *a glucosaminyltransferase [42]. Grigorian et al. [39] reported Vitamin D increasing MGAT1 expression and decreasing MS risk when combined with enhanced CTLA-4 N-glycosylation. This environment-gene interaction suggests a role for vitamin D in MS risk reduction [39].

Vitamin D status and VDR polymorphisms appear to correlate with the incidence and severity of autoimmune diseases [12]. Few polymorphisms have been studied in relation to autoimmune disease in general and only a limited number have been associated with MS specifically. Genetic studies on NMO are limited, however, a recent whole genome sequence study identifying variants in the MHC region suggests that NMO may be even more similar to SLE than to MS [43]. It may not be possible, therefore, to extrapolate findings from MS genetic studies and apply them directly to NMO which only emphasizes the need for more NMO research.

The human VDR gene spans approximately 75 kb on chromosome 12q13-14, composed of 11 exons with introns in between [44]. The four VDR polymorphisms most widely studied include: ApaI in intron 8 (A/C, rs7975232), BsmI in intron 8 (G/A, rs1544410), FokI in exon 2 (C/T, rs10735810) and TaqI in exon 9 (T/C, rs731236) [45]. ApaI and BsmI may possibly interrupt mRNA stability, disrupt splice sites or interfere with the regulatory elements of introns [46]. Fok I changes the start codon and causes a shortened VDR protein, and TaqI interrupts the stability of *VDR* mRNA [46]. The results of MS studies overall, however, have made it difficult to draw a solid conclusion about their roles.

A significant association is reported for MS and the FokI polymorphism in a region of Turkey [47]. However, no increase in risk of MS is reported for Asians and Caucasians with the VDR gene polymorphisms ApaI, BsmI, FokI, and TaqI [9]. In meta-analysis Tizoui et al. [13] were able to report a significant association between MS with *Apa*I polymorphism when codominant, and *Fok*I polymorphism in both dominant and codominant models. *Apa* and *Fok*II homozygote genotypes (AA and FF) were found to pose a high risk of MS but *Taq*I and *Bsm*I polymorphisms were not implicated [13]. It seems that VDR gene polymorphisms may also affect the severity and course of the disease. For example, the Fok-I “f” allele was linked to decreased level of disability 10 years after disease onset in the UK [48].

Results from various studies are clearly contradictory and reasons may include but are not limited to, small sample size, low statistical power, difference in ethnicities, geographic variation, interaction with other genetic or environmental factors and clinical heterogeneity [13].

The etiologies of autoimmune diseases such as NMO and MS are complex and polymorphisms may exert only a modest effect. Although *Apa*I, *Bsm*I and *Taq*I polymorphisms result in different stability or translational efficiency of RNA, they do not appear to affect VDR protein structure directly. *Fok*I polymorphism is an exception as it appears to affect both VDR protein structure and transcriptional activity [13, 46]. Overall, vitamin D exposure may be more important to consider than polymorphisms. Ponsonby et al. [49] illustrate this point well albeit regarding another type of autoimmune disease, type 1 diabetes mellitus. The study concluded that regional winter UV radiation levels determined the magnitude of association between VDR polymorphisms and disease. The association of T1DM with some alleles increased at higher winter UV radiation levels, while other allelic associations decreased [49]. This meta-analysis vividly portrays why it is necessary to consider a bigger picture when studying vitamin D, including environmental factors as opposed to viewing polymorphisms in isolation. Tizaoui et al. [13] meta-analysis also reported that latitude affects the association between *Apa*I polymorphism and MS risk.

Some relationships between genotype and disease depend on exposure level to an environmental factor. In other words, some associations are only apparent if there is high exposure while others are observed with lower exposure. One study hypothesized that an association of a *VDR *gene polymorphism with MS might only be penetrant in a population with a sufficient vitamin D status [48]. This is supported by the study of Handel et al. [50] which was conducted in vivo and able to correlate the amount of VDR binding with serum level of vitamin D. Autoimmune disease associated loci, and genes for T-regulatory and T-helper cells were enriched with VDR binding sites when vitamin D levels were greater than 75 nmol/L [50].

Considered together, the aforementioned studies suggest that vitamin D polymorphisms are probably important to the etiology of NMO and MS, but should not overshadow the potentially bigger problem of vitamin D deficiency. The issue of vitamin D deficiency is, however, further complicated by findings that there are some gene polymorphisms important for vitamin D function which predispose individuals to developing vitamin D deficiency [51], and possibly, autoimmune diseases such as MS [52]. Such single nucleotide polymorphisms (SNPs) have been identified within a Mendelian randomization study of MS patients and healthy controls near the following genes: *GC *(vitamin D-binding protein), *DHCR7 *(7-dehydrocholesterol reductase, enzyme catalyzing the conversion of cholesterol to vitamin D precursor), *CYP2R1 *(vitamin D-25-hydroxylase), and *CYP24A1*(1,25-dihydroxyvitamin D-24-hydroxylase, degrades vitamin D) [53]. These SNPs do not seem to display linkage nor are they associated independently with MS but they are associated with very low vitamin D levels [53]. When study groups are sorted based on the numbers of SNPs, Mokry et al. [53] reported that the polymorphisms have an additive effect, with the genetically determined vitamin D levels being inversely correlated with MS frequency. A 3.7-fold increased risk of MS appears to be associated with one *CYP24A1*-linked SNP alone [53]. A variant of *CYP24A1 *is purported to be associated with MS risk due to its increased transcription found along with frontal cortex inflammation. This finding suggests that increased CYP24A1-related vitamin D degradation may make the brain vulnerable for MS attack [54].

### 3. Vitamin D—Estrogen Interactions

Involvement of estrogen in vitamin D-related autoimmune disease seems to be logical in that women are at a higher risk than men for autoimmune diseases [4]. Being that women are characteristically vulnerable to autoimmune disease, it is plausible that a synergistic relationship between vitamin D and estrogen exists in the pathophysiology of MS and NMO.

Estrogen appears to be a modulator of CYP24A1 expression [55] and the synergistic relationship between estrogen and vitamin D activity might therefore be important to the etiology of demyelinating autoimmune diseases. Autoimmune diseases are more common in women than men [56], although, there is uncertainty as to why this sex-related disparity exists. One hypothesis suggests that because women may exhibit a stronger immune response than men, aggressive attacks on self-antigens are more likely [48]. Understanding this vulnerability requires the acknowledgment of estrogen's effects in the inflammatory process [57]. MS and NMO both target the CNS. Attention is therefore drawn to estrogen because it is shown to preserve myelin and other vital neurological components such as axons and synapses [58].

The role of estrogens in neuroprotective mechanisms has been illustrated in many studies. Precisely how the immunological effects of estrogens appear to be mediated specifically through vitamin D is of great interest, but not well studied. Both estrogens and vitamin D (cholecalciferol) are steroid hormones. These steroid hormones may work alongside each other in a synergistic relationship and it may be more helpful to view them as partners to better understand the mechanisms underlying autoimmune demyelinating diseases. Estrogen enhances VDR gene expression [59] and appears to inhibit vitamin D degradation via repression of CYP24A1 [55, 4]. Vitamin D, in turn, increases synthesis of estrogens by enhancing gene expression of aromatase, also known as estrogen synthase (CYP19) [60, 61].

The mechanisms by which estrogen increases the half-life of vitamin D and its responsiveness through VDR transcripts and CYP24A1 repression are unknown. A study investigating signaling pathways in breast and colon cancer suggest that E2 induces ERK 1/2 activation and transcriptional activity, which results in upregulation of expression of the VDR gene [59]. Spanier et al. [4] proposed an epigenetic mechanism for E2 decreasing the transcription of CYP24A1A in a study of rodent CD4+ T-cells. E2 is shown to recruit DNA methyltransferases to CpG-rich regions in promoters, thereby silencing genes by the mechanism of transcriptional repression [62]. CYP24A1 promoters are shown to be methylated in the exact same regions within human placental tissues [63]. Hence, a possible model that remains to be tested is E2 silencing CYP24A1 transcription promoters via methylation [4].

Vitamin D also upregulates aromatase mRNA in bone tissue which increases E2 levels and increases osteoblast and fibroblast activity for promoting bone mineralization [64]. Vitamin D may enhance aromatase in selective tissues outside of bone, thereby functioning as a selective aromatase modulator (SAM). A decrease in *CYP19* gene expression in VDR-null female rodents is associated with lower aromatase activity and lower circulating levels of E2 compared to wild-type rodents with intact VDR function [61]. Another study identified transcripts of *CYP19* and *HSD17B4* (encodes for 17*β*-hydroxysteroid dehydrogenase which interconverts estrone (E1) and estradiol (E2)) in splenic T lymphocytes of rodents [60]. These findings together suggest that T lymphocytes, important to the process of autoimmune disease, synthesize estrogens and may rely on the synergistic relationship with vitamin D.

As previously mentioned, the pathogenesis of experimental autoimmune encephalomyelitis (EAE), an animal model of MS, is increased by vitamin D deficiency and decreased by vitamin D supplementation, notably in a female-biased manner [4]. These findings suggest that cases of female MS, and perhaps NMO, might be preventable by vitamin D supplementation. More in depth exploration of this mechanism is needed to determine how the hormonal interplay can effectively be used as a risk-reduction strategy. It is noteworthy that when a major source of estrogen was removed via ovariectomy, the neuroprotective effects of vitamin D were no longer observed, but were restored with replacement of estrogen [4]. Notably, estrogen was not able to impart any resistance to EAE with CD4+ T cells which lacked the VDR [4], so it seems that estrogen may be a requirement for some vitamin D activity. Vitamin D also mediates the estrogen increase in T reg cells, possibly hinting to the event whereby women with insufficient vitamin D levels may lose self-tolerance and are left susceptible to autoimmune attack [4]. In that regard, one study reports that estrogen treatments alone offer protection against demyelination, axon loss, and CNS inflammation [65].

## 4. Summary and Conclusions

Epidemiological evidence points to global vitamin D deficiency, prevalent in all ages regardless of climate or geographical location [16]. Autoimmune diseases also appear to fall into this vitamin D-deficient category, hence, supplementation is being investigated and levels are monitored to discover correlations with disability.

Taken together, the findings of this review suggest that vitamin D supplementation has potential as a treatment for demyelinating autoimmune diseases. Existing studies, however, continue to present conflicting data regarding clinical significance. Determining whether vitamin D may even serve as prophylaxis for NMO and MS or even reduce disability/relapse rate still requires intense investigation. The role of vitamin D is so complex and the intricacies of its role in immunity depend on many factors. This creates some difficulty in formulating an ideal way to utilize vitamin D for its immunological protection. Hence a more detailed mechanism is needed and this review pinpoints VDR polymorphisms and estrogen as potential gateways for understanding how vitamin D mediates protection against demyelinating autoimmune diseases such as NMO and MS. Environmental interactions dictate VDR function to an extent and UV levels cannot be neglected when comparing and assessing vitamin D sufficiency. Women make up a considerable proportion of autoimmune disease diagnoses and studies are currently exploiting the synergistic relationship estrogen has with vitamin D to observe for clinical improvement. Each autoimmune disease has different pathological origin so observations may not be applicable across the board [48], but this does not diminish the impact that vitamin D may have. Epidemiological studies are not sufficient in isolation, requiring genetic and biological integration to capture the many role players in the immunoprotective mechanisms which vitamin D orchestrate.

While it may appear difficult, it should not be too long before we identify how to prevent the painful relapses and crippling disabilities that come about from immune systems gone haywire. A study is already hypothesizing VDR-activating drugs may be able to enhance remyelination in MS patients and other demyelinating diseases which may possibly include NMO [66]. If the immunological mechanism by which vitamin D exerts its effects is mapped out in more detail, a better understanding will be obtained to determine how to utilize it, whether as prophylaxis or maybe even just a marker of disease severity and warning sign of relapse on the horizon.
